# Association Study between Promoter Polymorphism of *TPH1* and Progression of Idiopathic Scoliosis

**DOI:** 10.1155/2016/5318239

**Published:** 2016-05-16

**Authors:** Vasil Yablanski, Svetla Nikolova, Evgeni Vlaev, Alexey Savov, Ivo Kremensky

**Affiliations:** ^1^Department of Orthopedics and Traumatology, Tokuda Hospital Sofia, 51B Nikola Vaptsarov Boulevard, 1407 Sofia, Bulgaria; ^2^National Genetic Laboratory, Medical University-Sofia, 2 Zdrave Street, 14th Floor, 1431 Sofia, Bulgaria; ^3^Molecular Medicine Center, Medical University-Sofia, 2 Zdrave Street, 14th Floor, 1431 Sofia, Bulgaria

## Abstract

The concept of disease-modifier genes as an element of genetic heterogeneity has been widely accepted and reported. The aim of the current study is to investigate the association between the promoter polymorphism* TPH1* (rs10488682) and progression of idiopathic scoliosis (IS) in Eastern European population sample. A total of 105 patients and 210 healthy gender-matched controls were enrolled in this study. The* TPH1* promoter polymorphism was genotyped by amplification followed by restriction. The statistical analysis was performed by Fisher's Exact Test. The results indicated that the genotypes and alleles of* TPH1* (rs10488682) are not correlated with curve severity, curve pattern, or bracing. Therefore, the examined polymorphic variant could not be considered as a genetic factor with modifying effect of IS. In conclusion, this case-control study revealed no statistically significant association between* TPH1* (rs10488682) and progression of IS in Eastern European population sample. These preliminary results should be replicated in extended population studies including larger sample sizes. The identification of molecular markers for IS could be useful for a more accurate prognosis of the risk for a rapid progression of the curve. That would permit early stage treatment of the patient with the least invasive procedures.

## 1. Introduction

Idiopathic scoliosis (IS) is known for centuries but its etiology still remains unclear.

The studies on IS genetics have indicated substantial heterogeneity in the etiology of the disease. First, there are predisposition genes that usually have low penetrance and are associated with a moderate increase of the risk for development of IS. In addition to predisposition to the development of deformity, genetic factors could also influence the curve progression [1]. The concept of disease-modifier genes as an element of genetic heterogeneity has been widely accepted and reported [1–3].

Melatonin is a focus of studies of the mechanism underlying the development of deformity and genes involved in melatonin signaling or biosynthesis are possible candidate-genes for IS.

Genetic variants of melatonin receptor 1A (*MTNR1A*) were not associated with IS, in a linkage study of 47 American families [4], nor in a larger Chinese female cohort (226 cases/277 controls) [5] or American cohort (589 cases/1,533 controls) [6].

Three polymorphic variants in the coding region of melatonin receptors 1B (*MTNR1B*) were not associated with IS, in Chinese females (473 cases/311 controls) [7]. Later the team extended the case-control study (a total of 814 cases/651 controls) and found an association between the promoter single nucleotide polymorphism (SNP) rs4753426 and curve predisposition [8]. This association was not further confirmed in two American studies (589 cases/1,533 controls [6] and 406 cases/479 control [9]), in Hungarian study (126 cases/197 controls) [10], in Bulgarian study (94 cases/188 controls) [11], and in Japanese study (798 cases/1,239 controls) [12].

Some components of the melatonin pathway not associated with IS included aralkylamine* N*-acetyltransferase (*AANAT*) in a Chinese study (103 cases/107 controls) [13] and in an American study (589 cases/1,533 controls) [6], G protein-coupled receptor 50 (*GPR50*) in the United States (406 cases/479 controls) [9], and acetylserotonin* O*-methyltransferase (*ASMT*), previously known as hydroxyindole* O*-methyltransferase (*HIOMT*) and protein kinase C delta (*PKCD*) in a separate American cohort (589 cases/1,533 controls) [6].

The promoter polymorphism* TPH1* (rs10488682 T/A) was associated with the genetic predisposition to the adolescent idiopathic scoliosis (AIS) in Chinese cohort study (103 cases/107 controls) [13], but this association was not confirmed in Japanese (798 cases/1239 controls) [12] or American population sample (589 cases/1,533 controls) [6]. A Chinese case-only study (90 cases/222 cases) found that patients with the polymorphic allele at the rs10488682 site of the* TPH1* gene are prone to be resistant to brace treatment [14].

Our previous study in Bulgarian population found no association between* MTNR1B* (rs4753426) and IS predisposition or curve progression [11]. The aim of the current study is to investigate the association between the promoter polymorphism* TPH1* (rs10488682) and progression of idiopathic scoliosis (IS) in Eastern European population sample.

In order to fulfill this aim, the following associations between the SNP and (i) curve severity (case-control study), (ii) curve pattern (case-only study), and (iii) brace treatment outcome (case-only study) were explored among Bulgarian patients.

## 2. Materials and Methods

A total of 105 patients and 210 healthy gender-matched controls were enrolled in this study. All participants were included only after the subjects or parents of the minor patients signed a written informed consent.

### 2.1. Patients

Patients with IS were recruited with the help of orthopaedic surgeons from Tokuda Hospital Sofia. Diagnosis was confirmed radiologically. The curves were measured by the Cobb method. Secondary scoliosis was excluded. The mean age at the beginning of the disease was 11.2 ± 3.1 years. Female (*n* = 86) and male (*n* = 19) patients were included.

For the aims of the case-control study the patients were divided into two groups according to the preoperative or the last follow-up Cobb angle measurement: progressive scoliosis (mean Cobb angle = 62.7 ± 17.4°) and nonprogressive or slowly progressive scoliosis (mean Cobb angle = 22.1 ± 6.3°). Then these groups of progressive (*n* = 84) and nonprogressive/slowly progressive scoliosis (*n* = 62) were compared to the control group. In this way the possible association between* TPH1* and curve severity was examined.

For the aims of the case-only study all cases were divided into three groups according to curve pattern, thoracic (*n* = 62), thoracolumbar (*n* = 31), and lumbar (*n* = 12) and then compared to each other. In this way the possible association between* TPH1* and curve pattern was examined. Additionally, progressive cases (*n* = 84) were separated into two groups, with (*n* = 49) and without (*n* = 34) previous brace treatment, and compared to each other. In this way the possible association between* TPH1* and brace treatment outcome was examined.

### 2.2. Controls

The control group including healthy subjects without clinical signs of IS was recruited from a pool of unrelated gender-matched volunteers from other units and clinics of Tokuda Hospital Sofia, National Genetic Laboratory, hospital staff members, and students. The controls were selected among adult patients with skeletal maturity with negative family history of IS. Radiological examination was not performed in the control group.

### 2.3. Genotyping

Genomic DNA was extracted from the peripheral blood leucocytes using magnetic bead technology (chemagic DNA Blood Kit special, Chemagen) on automated high throughput nucleic acid isolation platform (chemagic Magnetic Separation Module I, Chemagen).

The* TPH1* promoter polymorphism was genotyped by amplification followed by restriction.

The primer set consisting of forward primer 5′-AAGAAGTTGCACAATGCAGACA-3′ and reverse primer 5′-GTTGGGAAGACTGCAAGAAGC-3′ was used. The polymerase chain reaction (PCR) was carried out in a reaction mix of 20 *μ*L containing 100 ng DNA and 10x Prime Taq buffer (Genet Bio, Daejeon, Korea), 10 mM dNTPs Mixture (Genet Bio, Daejeon, Korea), 20 pmol forward and reverse primers (AlphaDNA, Montreal, Canada), and 0.1 U Prime Taq DNA Polymerase (Genet Bio, Daejeon, Korea). PCR amplification was performed in an AB 2720 Thermocycler (Life Technologies, NY, USA) with an initial denaturation at 94°C for five minutes and a final extension of seven minutes at 72°C. The following thermal cycle was repeated 30 times: denaturation at 94°C for 30 seconds, annealing for 30 seconds at 58°C, and extension at 72°C for 30 seconds.

The restriction fragment length polymorphism (RFLP) analysis was performed with the endonuclease* Spe*I (NEB, Ipswich, MA, USA), according to the manufacturer's instructions, and the restriction fragments were separated on agarose 3% gel in VG-SYS Horizontal Electrophoresis System (Biochrom, Miami, USA). The lengths of the fragments representing the genotypes are 260 bp (AA), 146 + 114 (TT), and 260 + 146 + 114 bp (TA), respectively.

### 2.4. Statistical Analysis

The statistical analysis was performed by Fisher's Exact Test to make genotype and allele comparisons between cases and controls as well as test for Hardy-Weinberg equilibrium. A value of *p* < 0.05 was considered to be statistically significant for comparison between data sets. Odds ratios (OR) were calculated with 95% confidence interval (95% CI). Statistical analysis was conducted with the IBM SPSS 19.0 (NY, USA) software package for Windows.

## 3. Results and Discussion

A case-control study and a case-only study to investigate the association between* TPH1* (rs10488682 T/A) and IS progression were conducted in Eastern European population.

Curve severity and curve pattern play a role in progression of IS. The general rules of progression are that larger curves carry a higher risk of progression than smaller curves, and thoracic and double primary curves carry a higher risk of progression than single lumbar or thoracolumbar curves [15]. We separated the cases in subgroups according to curve severity and then compared the genotype and allele frequencies in patient subgroups to the control group (case-control study). We also divided the cases in subgroups according to curve pattern and then compared the genotype and allele frequencies in the different subgroups of patients (case-only study) under genotypic (codominant, dominant, and recessive) and allelic model.

As a main nonoperative treatment for IS patients, brace treatment has proved to be effective in preventing curve progression by many studies conducted [16–18]. The knowledge of factors that affect the effectiveness of brace treatment is not clear enough to accurately predict its final outcome yet [14]. Risser sign and curve magnitude were found to be associated with failure of brace treatment [19]. Curve pattern has also been proposed to be a prognostic factor for brace treatment; nevertheless, the predictive value of different curve patterns varied in three independent series [19–21]. Xu et al. demonstrated that genetic factors could have an influence on the outcome of brace treatment. The authors concluded that the* ERα* and* TPH-1* genes are independent factors predictive of bracing effectiveness [14].

We also divided the cases in subgroups according to brace treatment outcome and then compared the genotype and allele frequencies in the different subgroups of patients (case-only study) under genotypic (codominant, dominant, and recessive) and allelic model.

Genotypes were in Hardy-Weinberg equilibrium. The genotype and allele frequencies of the* TPH1* promoter polymorphism were similar in the progressive group (Cobb angle >40°) compared to the control group (*p* > 0.05). In conclusion, the genotypes and alleles of* TPH1* (rs10488682 T/A) could not be associated with the curve severity of IS among Bulgarian patients. This result corresponds to the previous American population study (589 cases/1,533 controls) [6]. The major limitation of the current study is the small sample sizes that could affect the statistical power of the results. For the dominant genetic model (TT + TA versus AA) the statistical power is 54.6%. This means that we would need a sample of 175 per group to yield power of 80%. In the nonprogressive/slowly progressive group with Cobb angle <40° the genotype/allele frequencies were also comparable with the control group and the statistical power was about 20% that means we would need a sample of 211 per group to yield power of 80%.

Odds ratios of genotypes and alleles in the subgroups are summarised in Table 1.

The genotype and allele frequencies of the* TPH1* promoter polymorphism were comparable between the subgroups with different curve pattern (*p* > 0.05). On the basis of these results, the genotypes and alleles of* TPH1* (rs10488682 T/A) could not be associated with the curve progression of IS among Bulgarian patients. A Chinese case-only study found no significant differences of the genotype or allele distribution between the groups of patients with different curve pattern [14]. The optimal sample size includes about 503 cases per group to yield statistical power of 80%.

Odds ratios of genotypes and alleles are summarised in Table 2.

The genotype and allele frequencies of the* TPH1* promoter polymorphism were also comparable between the progressive cases with and without previous brace treatment (*p* > 0.05). In conclusion,* TPH1* (rs10488682 T/A) could not be associated with the brace treatment final outcome. The observed differences in the results between our study in Eastern European population group and the study in Chinese population sample [14] could be explained with differences in genotype and allele frequencies in various population groups. The optimal sample size includes about 336 cases per group to yield statistical power of 80%.

Odds ratios of genotypes and alleles are summarised in Table 3.

The results indicated that the genotypes or alleles of* TPH1* (rs10488682) are not correlated with curve severity, pattern, or bracing. Therefore, the examined polymorphic variant could not be considered as a genetic factor with modifying effect of IS. These preliminary results should be replicated in extended population studies including larger sample sizes.

## 4. Conclusions

In conclusion, this case-control study revealed no statistically significant association between* TPH1* (rs10488682) and progression of IS in Eastern European population.

These results could not exclude a role of this polymorphic marker in the etiopathogenesis of IS in other population groups.

The identification of molecular markers for IS could be useful for a more accurate prognosis of the risk for a rapid progression of the curve. That would permit early stage treatment of the patient with the least invasive procedures.

## Figures and Tables

**Table 1:** Odds ratios of genotypes and alleles of tryptophan hydroxylase 1 (*TPH1*) polymorphism rs10488682 (T/A) in subgroups with different curve severity.

Subgroup	Genotype, allele	*N* (case/control)	*p* value	OR [95% CI]
Progressive IS Cobb angle > 40° (*n*_1_ = 84, *n*_2_ = 210)	TT versus AA	5/17 versus 43/135	1	1.10 [0.37–3.22]
	TT + TA versus AA	41/75 versus 43/135	0.07	1.61 [0.97–2.69]
	TT versus TA + AA	5/17 versus 79/193	0.82	0.89 [0.31–2.54]
	T versus A	46/92 versus 122/328	0.16	1.34 [0.89–2.03]

Nonprogressive or slowly progressive IS Cobb angle < 40° (*n*_1_ = 21, *n*_2_ = 210)	TT versus AA	2/17 versus 11/135	0.62	1.71 [0.34–8.53]
	TT + TA versus AA	10/75 versus 11/135	0.48	1.54 [0.62–3.79]
	TT versus TA + AA	2/17 versus 19/193	0.64	1.47 [0.31–6.98]
	T versus A	12/92 versus 30/328	0.33	1.43 [0.70–2.90]

A value of *p* < 0.05 was considered to be statistically significant. OR indicates odds ratio; CI, confidence interval; *n*_1_, number of patients; *n*_2_, number of controls.

**Table 2:** Odds ratios of genotypes and alleles of tryptophan hydroxylase 1 (*TPH1*) polymorphism rs10488682 (T/A) in subgroups with different curve pattern.

Curve pattern	Genotype, allele	*N* (*n*_1_/*n*_2_)	*p* value	OR [95% CI]
Thoracic/(thoracolumbar + lumbar) (*n*_1_ = 62, *n*_2_ = 43)	TT versus AA	4/3 versus 32/22	1	0.92 [0.19–4.51]
	TT + TA versus AA	30/21 versus 32/22	1	0.98 [0.45–2.14]
	TT versus TA + AA	4/3 versus 58/40	1	0.92 [0.20–4.33]
	T versus A	34/24 versus 90/62	1	0.98 [0.53–1.80]

Thoracolumbar/(thoracic + lumbar) (*n*_1_ = 31, *n*_2_ = 74)	TT versus AA	1/6 versus 15/39	0.66	0.43 [0.05–3.91]
	TT + TA versus AA	16/35 versus 15/39	1	0.98 [0.45–2.14]
	TT versus TA + AA	1/6 versus 30/68	1	0.92 [0.20–4.33]
	T versus A	17/41 versus 45/107	1	0.98 [0.53–1.80]

Lumbar/(thoracolumbar + thoracic) (*n*_1_ = 12, *n*_2_ = 93)	TT versus AA	2/5 versus 7/47	0.58	2.69 [0.43–16.7]
	TT + TA versus AA	5/46 versus 7/47	0.76	0.73 [0.22–2.47]
	TT versus TA + AA	2/5 versus 10/88	0.18	3.52 [0.60–20.6]
	T versus A	7/51 versus 17/135	1	1.09 [0.43–2.78]

A value of *p* < 0.05 was considered to be statistically significant. OR indicates odds ratio; CI, confidence interval; *n*_1_, *n*_2_, number of patients.

**Table 3:** Odds ratios of genotypes and alleles of tryptophan hydroxylase 1 (*TPH1*) polymorphism rs10488682 (T/A) in the progressive group (with and without bracing).

Progressive group	Genotype, allele	*N* (*n*_1_/*n*_2_)	*p* value	OR [95% CI]
Previous bracing/without bracing (*n*_1_ = 49, *n*_2_ = 34)	TT versus AA	1/4 versus 26/17	0.15	0.16 [0.02–1.59]
	TT + TA versus AA	23/17 versus 26/17	0.83	0.88 [0.37–2.12]
	TT versus TA + AA	1/4 versus 48/30	0.15	0.16 [0.02–1.47]
	T versus A	24/21 versus 74/47	0.38	0.73 [0.36–1.45]

A value of *p* < 0.05 was considered to be statistically significant. OR indicates odds ratio; CI, confidence interval; *n*_1_, *n*_2_, number of patients.
